# The impact of urban flower meadows on the well-being of city dwellers provides hints for planning biophilic green spaces

**DOI:** 10.1038/s41598-025-16420-8

**Published:** 2025-08-30

**Authors:** Katarzyna Simonienko, Edyta Jermakowicz, Piotr Szefer, Karolina Wróbel, Urszula Suprunowicz, Urszula Cwalina, Agata Kostro-Ambroziak

**Affiliations:** 1 Forest Therapy Center Katarzyna Simonienko, Białystok, Poland; 2 Department of Plant Biology and Ecology, Faculty of Biology, University of Bialystok, Białystok, Poland; 3 Faculty of Science, University of South Bohemia, Ceské Budejovice, Czech Republic; 4 Czech Academy of Science, Institute of Entomology, Ceské Budejovice, Czech Republic; 5 The Włodzimierz Chętnicki Biological Science Club, Faculty of Biology, University of Bialystok, Białystok, Poland; 6 Doctoral School of University of Bialystok, Białystok, Poland; 7 Department of Biostatistics and Medical Informatics, Faculty of Medicine, Medical University of Bialystok, Białystok, Poland; 8 Department of Zoology and Genetics, Faculty of Biology, University of Bialystok, 15-245 Białystok, Poland

**Keywords:** Open urban greenspace, Urban flower meadows, Human well-being, Physical and mental health of city dwellers, Ecology, Psychology, Environmental social sciences

## Abstract

Living surrounded by greenery has a relaxing effect and reduces physiological and psychological symptoms of stress. In view of the exponential growth of the urban population and disconnection with nature, supporting the physical and mental health of city dwellers is a huge challenge nowadays. In this context, urban flower meadows (UFMs), a relatively new management strategy cultivated in many cities, can be a very important component of urban greenery, which support human well-being. We investigated the emotional reception of UFMs, taking into account the features of different types of UFMs and the socio-demographic characteristics of respondents. Our research shows that urban flower meadows evoke positive emotions regardless of the age, gender and place of origin of respondents. While some structural variables of UFMs, particularly the proportion of green in relation to other colours, the representation of various flower colours, the proportion of yellow flowers, and the presence of alien plant species—influence people’s perception. Fewer colours and the absence of alien plant species tend to shift perception towards less positive emotions. The dominance of yellow flowers evokes positive emotions. These results are helpful for the further planning of UFMs to better reinforce the well-being of all city dwellers.

**Supplementary Information:**

The online version contains supplementary material available at 10.1038/s41598-025-16420-8.

## Introduction

Living in an optimal ecological niche ensures well-being and health^1^. As far as evolutionary conditions are concerned, grassy landscapes, with the availability of trees, good visibility and high biodiversity in the form of forest stands and meadows, provide the best environmental context to support our health^2–5^. Many studies confirm that being surrounded by greenery has a relaxing effect and reduces the physiological and psychological symptoms of stress^6–9^, consequently providing protection from mental health problems in the future^10,11^. Contact with nature can also help in the recovery of physical and mental health^12^. Research conducted on huge populations shows that the annual incidence of 15 out of 24 distinguished disease groups was lower in green areas, especially in the case of anxiety and depression^13^. The importance of reconnecting children with nature is highlighted in many aspects of physical, mental, and environmental well-being^14^. It is believed that human health is strictly connected to the condition of the environment and the health of other non-human species, and together, they create the common, global health of the whole planet and its inhabitants. This conception has been named the ‘The One Health’ approach. It describes and expands cooperation and communication between various scientific disciplines at local, national and global levels in search of better health for all: people, animals and the ecosystem^15^. Supporting biodiversity and promoting the physical and mental health of city dwellers is a huge challenge nowadays, especially due to intensive urbanization^16,17^. Currently, more than half of the human population lives in cities, and it is estimated that by 2050, it will be 68%^18,19^. Research shows that an inability to observe and experience nature negatively impacts the general well-being of city dwellers^20^. People living in urban areas often appear physically and psychologically disconnected from nature. Scientists consider that this might result in suboptimal levels of hedonic and eudaimonic well-being^21^. Greenery in the city is of particular importance, on the one hand, protecting people from perceiving stressful stimuli, such as ‘concrete deserts’, intensive traffic, noise, and crowds; on the other—inducing relaxation on a conscious and unconscious level, involving the central and autonomic nervous system^22^. Well-designed urban green areas may have multiple benefits for residents’ health, improve social relations, and support biodiversity^23,24^. WHO, approving the Healthy Cities Movement, indicates that green areas in the city support a friendly environment, as well as the mental health of residents, by stabilising emotions and reducing stress. Lee and Maheswaran^25^ pointed out a causal link between various mental health indicators and urban green space. City dwellers living in a fairly green environment have lower than average stress levels and higher life satisfaction^26,27^. It has been confirmed in several studies that living in green areas, especially in cities, is associated with fewer prescriptions of antidepressant drugs in adults and children^28,29^, as well as less suicide^30^, general mortality and violence^31^. The positive impact of urban green areas on mental well-being depends on the amount of green areas around^32^. Rahimi-Ardabili et al.^33^ showed even more benefits resulting from the proximity of greenery, including mental health, overall health, optimal body weight and more favourable cardio-metabolic and cerebrovascular outcomes. Living in green spaces in childhood correlates with a lower incidence of depression in adulthood, and this correlation was stronger in densely populated areas^34^.

According to Hartig’s theory, natural visual stimuli are important for recovery from stress, and stress reduction happens faster in nature than in urban settings^35^. There is a global interest in increasing the complexity of urban ecosystems to benefit both people and nature in cities^36^. The beneficial impact of urban parks, especially those with old-growth trees, on urban biodiversity and resident well-being is often emphasised in the literature^37–40^. In the case of open urban green spaces, their recreational function is highlighted in the context of the perception of the residents^41^. Introducing species-rich herbaceous communities, known as urban flower meadows (UFMs), has introduced a new quality, providing a win–win strategy for biodiversity and people^42^. They constitute one of the most valuable ecological models for the management of open urban greenery, which certainly supports biodiversity in cities by creating abundant food resources and shelter for invertebrates and vertebrates, positively influencing bioretention and reducing air pollution^43–47^, as well as leading to lower environmental and economic costs than lawn management^48^. Urban flower meadows were initially created as lawns, whose mowing was reduced due to periods of drought in the cities^49^. Furthermore, flowering plants, both native and alien to regional flora, were also sown and created specific urban flower meadows subjected to limited seasonal cutting, producing more structurally complex and florally diverse habitats^44,46,49^. UFMs also represent a kind of cultural element of continuity between natural and anthropogenic landscapes, have aesthetic value and can help people interact with nature and support a sense of social responsibility towards nature in line with the concept of reconciliation ecology^42,50^. It should be emphasized that there is a degree of flexibility concerning the size, location and plant species composition of urban flower meadows^51^. The latter leads to differences in UFM structure (e.g., number of colours, presence of alien plant species), which may modify the perception of this urban greenery. The impact of different types of nature on well-being also depends on social and cultural variables^52–54^ including people’s age and gender^55^, area of residence^56^ and socioeconomic status^57^. Thus, understanding how UFMs can be optimized to better support the well-being of all city dwellers is essential for the further planning of these urban open green areas.

The main objective of this study was to investigate the emotional reception of urban flower meadows (UFMs) by respondents and to find variables that influence it. We hypothesised that:

### H1

The presence of urban flower meadows improves well-being, evoking positive emotions.

### H2

The number of flowering plants, flower colours, and the percentage of greenery affect the perception of UFMs.

### H3

The naturalness of urban flower meadows, characterised by the presence of native flowering plant species, fosters positive emotions.

### H4

The perception of different urban flower meadows depends on the socio-demographic characteristics of the respondents. Thus, we asked: (1) Do different age groups rate emotions differently? (2) Do respondents living in the city and rural areas rate emotions differently? (3) Does respondent origin affect ratings? (4) Is there any interaction between origin and place of residence when it comes to assessment?

## Material and methods

### The urban flower meadow perception survey

**Fig. 1:**
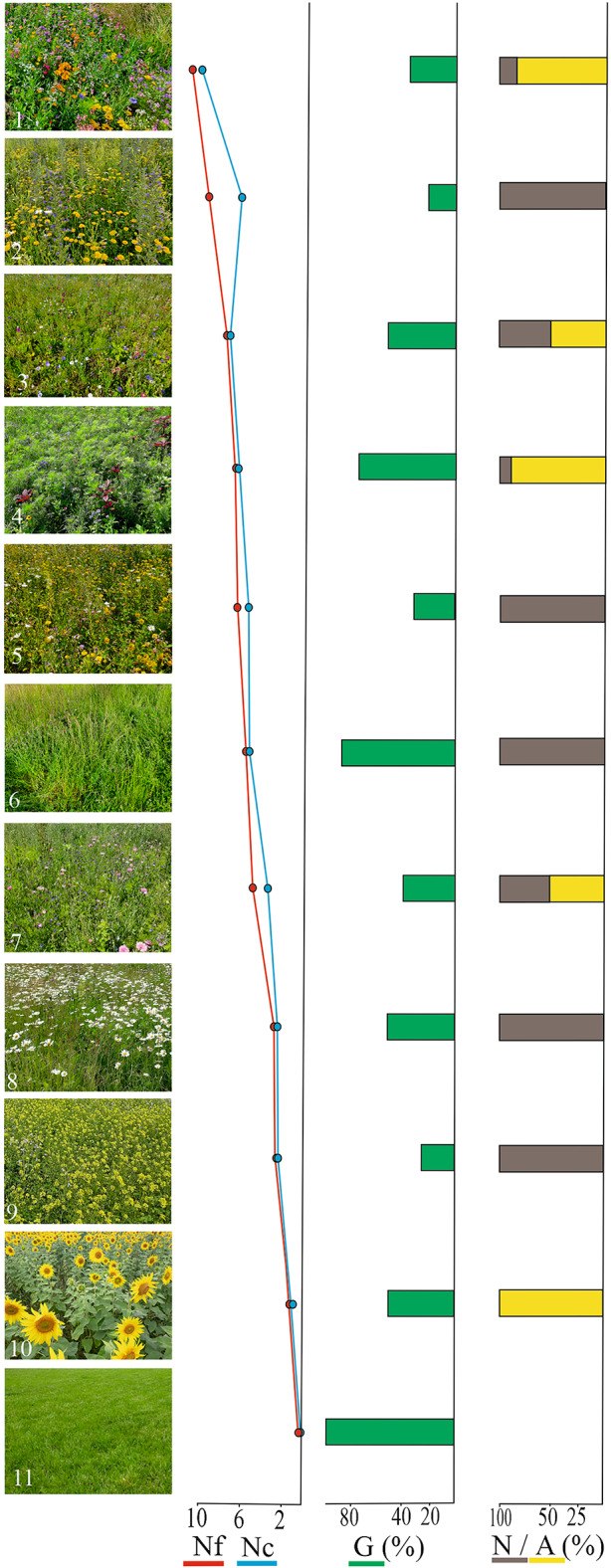
A set of photographs that were evaluated by respondents. Photos 1–9—urban flower meadows, 10—sunflower planting, 11—short-cut lawn. The presentation of UFM diversity: Nf—number of flowering plants, Nc—number of flower colours, G—percentage of greenery, N/A—proportion of native to alien plant species.

An internet survey was conducted to determine the impact of different types of urban flower meadows on their subjective reception. The survey consisted of 11 photographs, including nine different urban flower meadows, diverse in terms of vegetation (photo numbers 1–9), one sunflower planting (photo number 10) and one short-cut lawn (photo number 11); the latter used as an outgroup (Fig. 1). Meadows were selected among UFMs occupying an area of about 65,000 m^2^ in Białystok, in 2021, and represented different types of UFMs also cultivated in other cities in central Europe^51^. The examined urban flower meadows exhibited variations in internal structure, mainly in terms of plant species composition, which influenced the colourful nature and natural character of the sites. Plant species richness was assessed based on a list of flowering plant species determined during field studies. We chose flowering plants from the list of plant species compiled during the field visit, which were distinguishable in the photograph. Then we analysed a variable showing the number of flowering plants [Nf]. The number of flower colours [Nc] was determined directly from the images, as was the percentage of greenery [G]. The number of flowering species identifiable on photos ranged from 2 to 10, and the number of flower colours ranged from 1 to 9. The percentage of greenery ranged from 20 to 100% (Fig. 1). Additionally, we selected the yellow flower colour [Y] as the colour that differentiates the UFMs. Furthermore, a variable determining the domination (1) of yellow-coloured flowers [Y ≥ 50%] of all flower colours visible on the photographs, or lack (0) of domination [Y < 50%] of flower colours, was assessed (Fig. 1). The naturalness of flower meadows was determined according to the percentage of native [N] (including native plant species but also archeophytes established in Europe before 1492) and alien [A] plant species which are primarily garden and bedding species, not established in the native flora^58^. The percentage values were changed to a nominal scale where [A = 0] means no alien plant species, and [A = 1] indicates the domination of alien plants when alien plant species were ≥ 50% of all those visible on photos of flowering plant species. The respondents were not informed about the origin of the plants. Thus, we used this data to assess subconscious perceptions and preferences. In five instances, such species dominated or co-dominated [A = 1], while the remaining UFMs were natural in character, without alien species of flowering plants [A = 0].

Photographs of selected urban flower meadows were presented to internet respondents. Using the semantic differential scale (SD)^59^, the respondents rated the photos and specified their level of agreement with a statement by indicating a position along a continuous line between two endpoints on a 10-point scale in seven pairs of positive–negative emotions: (1) joyful–sad, (2) calm–disturbing, (3) safe–dangerous, (4) interesting–boring, (5) refreshing–tiring, (6) comfortable–uncomfortable, (7) natural–artificial. The respondents were also asked about their socio-demographic characteristics: (i) place of origin and (ii) current place of residence (groups for (i) and (ii): villages and towns of 1,000–5,000 inhabitants [1], cities of 5,000–50,000 inhabitants [2], cities of 50,000–250,000 inhabitants [3] and cities of > 25,000 inhabitants [4]), (iii) age (groups: 18–28 years [1], 29–39 [2], 40–50 years [3], 51–60 years [4] and 62–72 years [5]) and (iv) gender.

All experimental protocols were approved by The Medical University of Bialystok Code of Ethics for Research Workers (decision no. APK.002.287.2021). All methods were carried out in accordance with relevant guidelines and regulations (https://www.umb.edu.pl/en/s,20777/The_Code_of_Ethics_for_Research_Workers; Commission for Research Integrity, 2020). Informed consent was obtained from all subjects.

### Statistical analysis

We used two statistical approaches to examine respondent evaluation of the urban flower meadows. First, we performed PCA on the ratings to create a unique gradient of emotions. The axis was then used as the response variable in further analyses. Next, we constructed a linear mixed effects model with the PCA axis as a response variable and the main characteristics of each photographed meadow as explanatory variables. Respondents’ ID was used as a random effect, as a single person rated all 11 photos for each of the seven pairs of emotions. We created a series of models to test our main hypotheses. For the hypothesis encompassing urban flower meadow diversity (H: 1–3), we eventually used four of the most significant characteristics of meadow structure: A, G, Nc and Y. In the last model, we also included age, gender, origin and place of residence, and their interaction. To test the latter term’s significance, we performed a chi-square test for the model with and without the interaction term. In the fourth hypothesis (H: 4), we used simple LME models with only a single predictor (for questions 1–3 in the 4th hypothesis) or with their interaction (the last question in the 4th hypothesis).

In our second approach, we analysed each of the seven pairs of emotions studied separately using a five-point ordinal scale: very positive (for 1–2), quite positive (for 3–4), neutral (for 5–6), quite negative (for 7–8), very negative (for 9–10). For the individual emotions, we tested only for the significance of the interaction term, as in hypothesis four above (the last question). Due to unbalanced data from groups relating to place of origin and current place of residence, two categories were approved for statistical analysis: rural areas (including group [1] from the survey) and cities (including groups [2–4] from the survey). We used the Cumulative Link Mixed Model with the ID of respondents as a random factor using the *ordinal* library^60^. All analyses were performed using R Statistical Software^61^.

## Results

A total of 189 participants from different places in Poland, representing all the designated categories, took part in the survey.

### Reception of different urban flower meadows

**Fig. 2:**
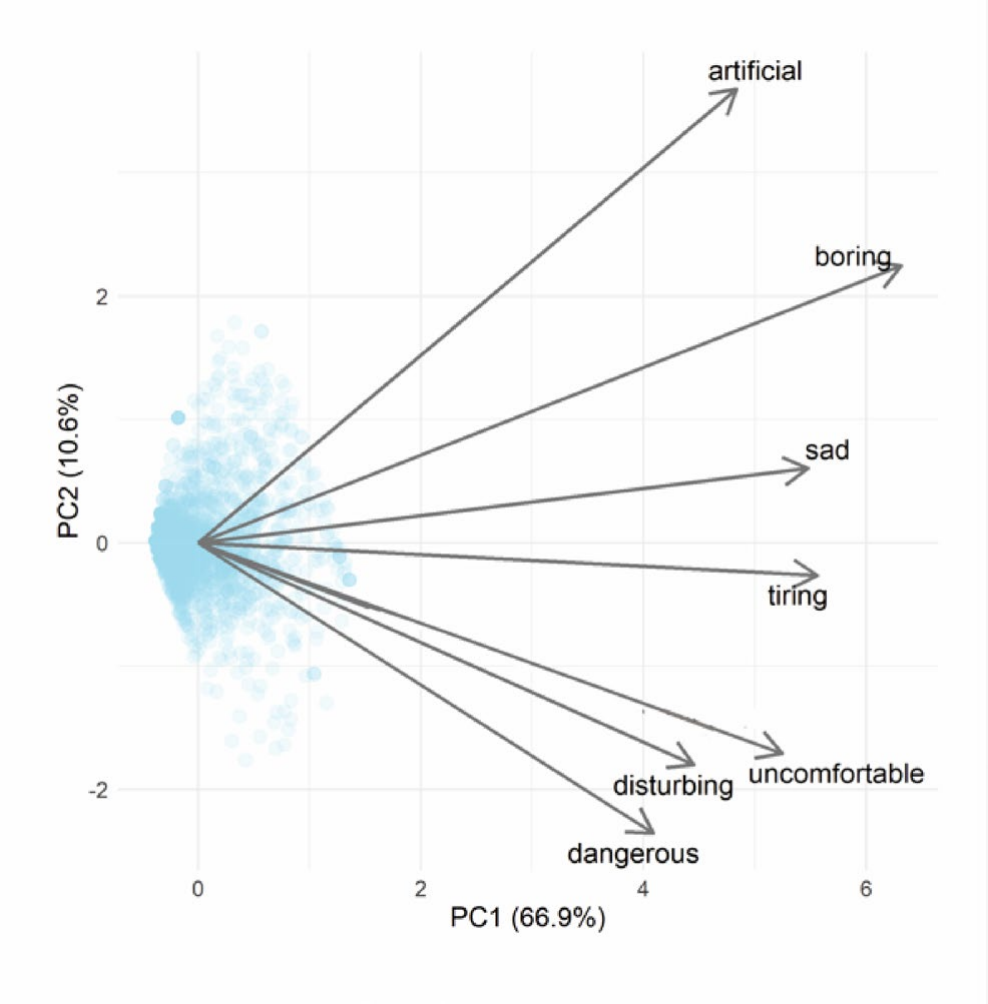
Principal Component Analysis (PCA) biplot visualising the relationship between samples (blue points) and contributing individual pairs of emotions (grey arrows). The first two principal components (PC1 and PC2) are shown, with axis labels indicating the percentage of variance explained. Variables are represented as vectors, where the direction and length indicate their influence on the principal components. Sample points are positioned based on their scores along these components. This analysis models the first principal component, approximating a positive-to-negative emotion gradient.

**Fig. 3:**
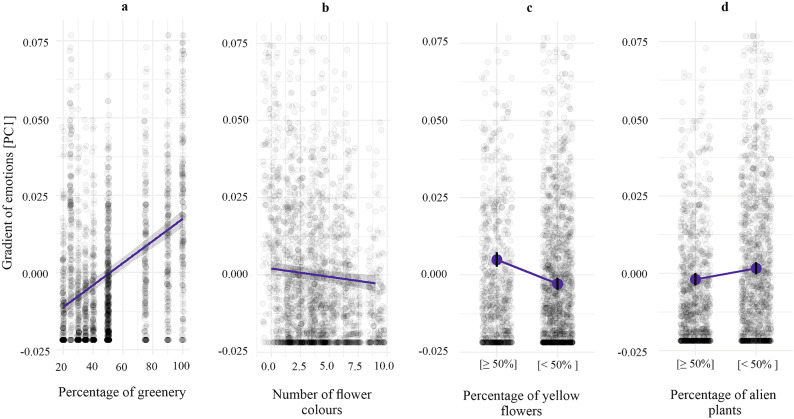
Partial correlation plots showing the relationship between the gradient of emotions (PC1) and four significant predictors, as evaluated using a Cumulative Link Mixed Model (CLMM). The model includes respondents as a random effect. The predictors shown are: (**a**) Percentage of greenery [G], (**b**) Number of flower colours [Nc], (**c**) Percentage of yellow flowers [Y], and (**d**) Percentage of alien plants [A]. The grey rings represent individual observations, the blue lines indicate model-predicted effects, and the shaded areas (**a**, **b**) and whiskers (**c**, **d**) show confidence intervals.

All examined UFMs were positively received by respondents (Fig. 2). The first PCA axis explained ~ 67% of the variation in the ratings. We found that urban flower meadow characteristics were a significant predictor of their perception. A higher percentage of greenery in relation to colours and the absence of alien plant species in urban flower meadows tended to shift the perception from the positive towards the more negative emotion in each of the studied pairs of emotions. A higher number of flower colours and the dominance of yellow flowers shifts the perception towards the more positive emotion in each of the emotion pairs (Table 1, Fig. 3). None of the respondent characteristics were significant when we used the simple LME models with single predictors (H: 4) (Table 1).

**Table 1:** Results of the linear mixed-effects model predicting the scores of the emotional gradient.

Predictors	Estimates	CI	p
(Intercept)	− 0.0143	− 0.0187 to− 0.0099	** < 0.001**
Age [2]	0.0004	− 0.0045 to 0.0054	0.871
Age [3]	− 0.0015	− 0.0068 to 0.0039	0.588
Age [4]	0.0010	− 0.0050 to 0.0070	0.743
Age [5]	− 0.0032	− 0.0162 to 0.0098	0.625
Percentage of greenery [G]	0.0004	0.0003 to 0.0004	** < 0.001**
Place of origin [rural areas]	0.0007	− 0.0037 to 0.0050	0.756
Number of flower colours [Nc]	− 0.0005	− 0.0009 to − 0.0002	**0.004**
Percentage of yellow flowers [Y < 50%]	− 0.0079	− 0.0102 to − 0.0055	** < 0.001**
Place of residence [rural areas]	0.0039	− 0.0009 to 0.0088	0.113
Gender [male]	0.0005	− 0.0036 to 0.0047	0.798
Percentage of alien plants [A < 50%]	0.0036	0.0019 to 0.0052	** < 0.001**
Random Effects			
σ^2^	0.0003		
τ00 person.id	0.0001		
ICC	0.3346		
N person.id	188		
Observations	2068		
Marginal R^2^/Conditional R^2^	0.164/0.443		

Fixed effects include socio-demographic factors, urban flower meadow characteristics and other relevant predictors. The estimates represent the direction and strength of each effect, with confidence intervals (CI) indicating uncertainty and p values (p < 0.05 statistically significant). The intraclass correlation coefficient (ICC) = 0.33 suggests moderate variation attributable to individual differences. The marginal R^2^ (0.164) represents variance explained by fixed effects, while conditional R^2^ (0.443) includes both fixed and random effects.

Significant values are in bold.

### Individual emotions

The results of the gradient model are also reflected in the individual pairs of emotions. Only in the joyful–sad, calm–disturbing and refreshing–tiring pairs of emotions was a significant effect found on the interaction between respondent origin and place of residence. Respondents with a city as their place of origin who also live in a city and those from rural areas who live in a city perceive urban flower meadows more positively. Regardless of origin, people who live in rural areas generally perceive UFMs less positively. The interactions between current place of residence and place of origin do not strongly influence the emotional perception of UFMs (see Supplementary Tab. S1 online). In the model without interaction, place of origin was not significant, and place of residence increased the probabilities of transitioning from positive to less positive emotions in the case of the joyful–sad, calm–disturbing, and refreshing–tiring pairs of emotions. There was no effect of gender. In the case of certain emotions, the age of the respondents had an impact on the perception of UFMs: meadows were perceived as much safer in the age group 40–50 years old and as more artificial in the age group 29–39 years old.

All four characteristics of the meadows had similar effects on the perception of UFMs (see Supplementary Tab. S2 online). A higher percentage of yellow flowers leads to more positive emotions in contrast to a higher percentage of greenery, which shifts perception towards the more negative emotions in all pairs of emotions. The UFMs with more colours were assessed towards the positive emotion in joyful–sad, safe–dangerous, interesting–boring, refreshing–tiring, and natural–artificial pairs. However, many colours shifted perception from ‘calm’ to ‘disturbing’. The number of colours was not significant for the comfortable–uncomfortable pair of emotions. The absence of alien plants shifts the perception into the less positive emotions in some of the pairs, specifically joyful–sad, interesting–boring, refreshing–tiring, and comfortable–uncomfortable. The higher proportion of alien plants in UFMs lead these UFMs to be perceived as more natural in the artificial–natural emotion pair.

## Discussion

The possibility of interacting in nature is an important factor supporting health and well-being, especially in the case of urban dwellers^12,25,62,63^. Although low-mown green spaces are still the dominant form of urban green space^64^, alternatives, such as urban flower meadows (UFMs), are gaining increasing popularity in the cities of Europe^42,65,66^. As our research shows, this kind of urban greenery evokes positive emotions in people, which improve their well-being. Southon et al.^42^ revealed that meadows were preferred over other green areas, including typical flower beds and mowed lawns. The creation of meadows improved the quality of the sites, which was noticed and appreciated, especially if people had information about their biodiversity potential and the cost savings due to the lack of frequent mowing^42^. However, certain characteristics of urban flower meadows significantly modify their perception by respondents. One particularly important feature turned out to be the percentage of greenery in relation to the other colours, representing diverse flowering plants. A higher proportion of green with fewer other colours tended to shift the perception towards the less positive emotion in each of the studied pairs of emotions. It is widely known that colors have a strong impact on human emotions and feelings^61,67,68^ but the psycho-physiological effects of plant colours depend on the location and kind of vegetation we see. Taking indoor environments into account, green plants lead to a feeling of comfort and high concentration, plants with white and yellow flowers make a place more pleasant, and red flowers create a feeling of luxury^69^. Regarding preferences in street plantings, people preferred flowers with bright colors^70^. In our study a dominance of yellow flowers shifts the perception of an UFM towards more positive emotions (more joyful, relaxing, safe, interesting, refreshing, and comfortable), similar to orange flowers in study of Zhang et al.^71^. This observation is consistent with research indicating that looking at yellow and red flowers indoors causes physiological relaxation, a higher sense of cheerfulness and comfort, and stress relief by significantly increasing parasympathetic nerve activity, improving overall mood status^72^. Interestingly, we also observe that multicolour meadows evoke disturbing rather than calming perceptions. This is also an important issue for the further study of UFMs, especially in the context of people who are sensitive to visual stimuli.

Why do simple green areas evoke less positive emotions than colourful ones? We hypothesise that in our survey, a higher proportion of the colour green could be perceived as less biodiverse and thus less supportive. People strongly prefer higher plant species richness in urban green spaces (i.e., parks, wastelands, streetscapes) and relate this diversity to their well-being^54,73–75^. Respondents do not notice the actual diversity of species (underestimating species richness), but they notice changes in native flower richness in those gardens where advertisements and public involvement were organised^74^. This is consistent with our results showing that the absence of alien species leads to rating an UFM as more natural. However, the absence of alien plants on studied meadows causes a less positive perception of UFMs, shifting emotions towards sad, boring, tiring and uncomfortable. This may result from the fact that the lower contribution of alien plants, which were ornamental, garden species, leads to less colourful urban flower meadows. This means that the aesthetic value of plants is more important for psychological well-being than whether they are perceived as more or less nativel. Hussain et al.^76^ showed that apparent health benefits, like stress reduction, were higher during participants’ direct contact with cultivated meadows than in unmanaged, more natural ones. Also, the attractiveness of the surrounding landscape and recreational usefulness were rated higher when visiting cultivated meadows^76^.

Including differences due to socio-demographic characteristics is critical for the successful planning of biophilic spaces, which reinforce the well-being of all city dwellers^55,77–79^. Both past childhood and current nature experiences and duration of those experiences significantly influence perception of nature^78^. Our findings indicate that the factors influencing the perception of urban flower meadows are mainly associated with place of origin and residence. People who lived in rural areas, regardless of their origin, perceived urban meadows less positively—less joyful, calming and refreshing than the city dwellers. Does this mean that city residents appreciate nature more, or on the contrary—they have less contact with it, so observing something that seems more common to people living daily in similar surroundings becomes a pleasant surprise? Maybe people who live closer to wild nature in rural areas have different wilderness standards, and city meadows seem more like a substitute for natural landscape, causing less enthusiasm. This may be related to the nature deficit syndrome^80^ and to the phenomenon of shifting baseline syndrome^81^. Both of these phenomena indicate that disappearing biodiversity leads to less human contact with nature and that requirements and expectations regarding what the natural environment should be are shifting. Subsequent generations set the bar lower and lower, considering poorer biodiversity as their starting point. Unsurprisingly, if expectations and norms for nature are lower in highly urbanised areas, without the possibility of experiencing nature and natural processes outside the city, then urban flower meadows, even with a less natural character, may arouse greater enthusiasm.

In our research, flower meadows were positively perceived by all age groups, but regarding some emotions, the age of the respondents modified the perception of UFMs. People aged 40–50 for whom urban flower meadows evoke more positive emotion connected them with safety, while respondents aged 29–39 tended to see them as more artificial than natural. The studies emphasize that the regular exposure to greenery lead to reduction of stress levels and an improvement in cognitive functioning among the elderly. They feel safe in these places and experience less loneliness due to social interactions, which in turn enhances their overall well-being^23,82^. On the other hand, it was shown that green areas are significantly less attractive to the group of younger people (18–31 years) than they are to the older age group. Young respondents tend to spend their free time online rather than in urban green areas, or may think that the city greenery is a place more for children or older people and see them as unattractive^83,84^. Experiences of the natural environment are often associated with some small acceptable discomforts, which can be considered ‘normal’ and safe, while for people who do not come into contact with them on a daily basis they are already associated with experiencing discomfort and a sense of tension, because it is a more alien and unusual environment^70^. It is important to note that the urban green space preferences of young adults are linked to physical activities in addition to environmental features^85^. In contrast, older adults tend to value the naturalness, calmness, and aesthetic qualities of green spaces^86^, which can provide urban flower meadows.

## Conclusion

Our research shows that urban flower meadows positively affect the well-being of all respondents regardless of age, gender and place of origin. Although UFMs evoke positive emotions, certain features of this urban greenery significantly modify their perception by respondents. Colourful UFMs, reflecting plant richness but not with too many colours, and dominated by yellow flowers, are the most desirable and should be promoted by managers of urban green infrastructure. Because people prefer higher plant species richness, which supports the biodiversity of many animals, including bees, UFMs are an example of a potential win–win situation in urban area management for humans and wildlife.

## Supplementary Information

Below is the link to the electronic supplementary material.

Supplementary Material 1

## Data Availability

All data generated or analysed during this study are included in this published article and its supplementary information files. Additional data will be made available from the corresponding author on request.
