# Supplementary material for: The impact of urban flower meadows on the well-being of city dwellers provides hints for planning biophilic green spaces
Source: Sci Rep. 2025 Aug 30;15:31981. doi: 10.1038/s41598-025-16420-8 (PMC12398554; doi:10.1038/s41598-025-16420-8)
Supplement: Supplementary file 1 — Supplementary Material 1 [file 41598_2025_16420_MOESM1_ESM.pdf]

Supplementary information

The impact of urban flower meadows on the well-being of city dwellers provides hints for planning biophilic green spaces

Katarzyna Simonienko, Edyta Jermakowicz, Piotr Szefer, Karolina Wróbel, Urszula Suprunowicz, Urszula Cwalina, Agata Kostro-Ambroziak*

Table S1 Model comparison results for the Cumulative Link Mixed Models (CLMM) evaluating the effect of current place of residence (village or city) and place of origin (village or city) on the probabilities of moving from a low to a higher score (from positive to negative emotions). Each pair of emotions includes results for two models: the Full Model (without interaction) and the Interaction Model (including interaction between place of residence and place of origin). Model performance is assessed using Akaike Information Criterion (AIC) and log-likelihood (log-Likelihood) values. The Likelihood Ratio (LR) statistic and Chi-square test (Pr(>Chisq)) evaluate whether adding the interaction term significantly improves model fit. None of the interaction models show significant improvement (p > 0.05), suggesting that interactions between place of residence and place of origin do not strongly influence the emotional gradient.

Pair of emotions	Model Type	AIC	Log -Likelihood	LR Statistic	df	Pr(>Chisq)
joyful – sad	Full Model	4355.2	-2161.6	-	-	-
	Interaction Model	4356.6	-2161.3	0.5431	1	0.4611
calm – disturbing	Full Model	4222	-2095	-	-	-
	Interaction Model	4223.7	-2094.9	0.2077	1	0.6485
safe – dangerous	Full Model	4260.9	-2114.5	-	-	-
	Interaction Model	4262.4	-2114.2	0.5077	1	0.4761
interesting – boring	Full Model	4640.5	-2304.3	-	-	-
	Interaction Model	4642.5	-2304.2	0.0235	1	0.8781
refreshing – tiring	Full Model	4475.6	-2221.8	-	-	-
	Interaction Model	4477.4	-2221.7	0.2495	1	0.6174
comfortable – uncomfortable	Full Model	4664.3	-2316.2	-	-	-
	Interaction Model	4666.2	-2316.1	0.1465	1	0.7019
natural – artificial	Full Model	4246	-2107	-	-	-
	Interaction Model	4248	-2107	0.0741	1	0.7855

Table S2 Results of the Cumulative Link Mixed Model (CLMM) for each of the studied pairs of emotions.

The table presents odds ratios (ORs), 95% confidence intervals (CI), and p-values for key predictors influencing emotional gradient scores. Higher ORs (>1) indicate an increased likelihood of transitioning from very positive to a more negative emotional category, while lower ORs (<1) indicate a decreased likelihood.

Fixed effects include demographic variables (age, gender, place of origin, current place of residence), urban flower meadow characteristics (proportion of greenery, number of flower colors, proportion of yellow, alien plant presence), and seven pairs of emotions: (1) joyful – sad, (2) calm – disturbing, (3) safe – dangerous, (4) interesting –boring, (5) refreshing – tiring, (6) comfortable – uncomfortable, (7) natural – artificial. Random effect variance estimates (τ00) reflect variability at the individual level, with Intraclass Correlation Coefficients (ICC) quantifying the proportion of variance explained by individual differences. Marginal R^2 represents variance explained by fixed effects, while Conditional R^2 includes both fixed and random effects.

	**JOUFUL – SAD**			**CALM – DISTURBING**			**SAFE – DANGEROUS**			**INTERESTING – BORING**			**REFRESHING – TIRING**			**COMFORTABLE – UNCOMFORTABLE**			**NATURAL – ARTIFICIAL**		
Predictors	Odds Ratios	CI	p	Odds Ratios	CI	p	Odds Ratios	CI	p	Odds Ratios	CI	p	Odds Ratios	CI	p	Odds Ratios	CI	p	Odds Ratios	CI	p
very positive|quite positive	1.89	1.26 – 2.83	**0.002**	1.46	0.91 – 2.35	0.121	0.70	0.43 – 1.16	0.170	1.19	0.78 – 1.82	0.427	1.14	0.72 – 1.78	0.580	0.73	0.46 – 1.16	0.184	1.14	0.68 – 1.90	0.628
quite positive|neutral	7.43	4.89 – 11.28	**<0.001**	7.33	4.50 – 11.94	**<0.001**	4.12	2.48 – 6.84	**<0.001**	4.59	2.97 – 7.08	**<0.001**	4.71	2.98 – 7.44	**<0.001**	3.32	2.08 – 5.31	**<0.001**	4.23	2.52 – 7.11	**<0.001**
neutral|quite negative	24.60	15.87 – 38.14	**<0.001**	25.48	15.32 – 42.36	**<0.001**	18.48	10.87 – 31.43	**<0.001**	12.27	7.86 – 19.15	**<0.001**	17.68	11.00 – 28.41	**<0.001**	12.08	7.44 – 19.62	**<0.001**	9.18	5.43 – 15.53	**<0.001**
quite negative|very negative	57.03	35.92 – 90.54	**<0.001**	60.48	35.27 – 103.73	**<0.001**	61.45	34.34 – 109.99	**<0.001**	30.57	19.27 – 48.51	**<0.001**	44.68	27.09 – 73.70	**<0.001**	33.82	20.24 – 56.51	**<0.001**	20.98	12.23 – 35.98	**<0.001**
Age [2]	1.06	0.66 – 1.69	0.822	1.05	0.59 – 1.88	0.866	0.64	0.34 – 1.20	0.166	0.93	0.55 – 1.55	0.770	0.93	0.54 – 1.62	0.806	0.88	0.49 – 1.57	0.671	2.35	1.23 – 4.50	**0.010**
Age [3]	1.01	0.61 – 1.68	0.963	0.89	0.47 – 1.68	0.718	0.49	0.24 – 0.97	**0.040**	0.80	0.46 – 1.41	0.449	0.78	0.43 – 1.42	0.420	0.61	0.33 – 1.15	0.128	1.89	0.93 – 3.83	0.078
Age [4]	0.92	0.52 – 1.65	0.786	1.18	0.58 – 2.41	0.652	0.79	0.37 – 1.72	0.557	0.91	0.48 – 1.71	0.769	0.71	0.36 – 1.41	0.328	0.90	0.44 – 1.81	0.759	2.18	0.98 – 4.82	0.056
Age [5]	0.64	0.18 – 2.24	0.481	0.76	0.16 – 3.65	0.729	0.89	0.17 – 4.52	0.884	0.54	0.13 – 2.15	0.380	0.64	0.15 – 2.78	0.552	0.92	0.20 – 4.15	0.913	1.42	0.26 – 7.80	0.687
Percentage of greenery [G]	2.83	2.45 – 3.26	**<0.001**	1.72	1.49 – 1.99	**<0.001**	1.93	1.67 – 2.23	**<0.001**	2.84	2.47 – 3.26	**<0.001**	2.30	2.00 – 2.65	**<0.001**	2.00	1.74 – 2.30	**<0.001**	2.16	1.87 – 2.50	**<0.001**
Place of origin [rural areas]	1.06	0.70 – 1.60	0.783	1.12	0.67 – 1.86	0.674	1.12	0.64 – 1.94	0.693	0.95	0.60 – 1.49	0.812	0.95	0.59 – 1.54	0.838	1.16	0.70 – 1.92	0.573	1.42	0.80 – 2.51	0.229
Number of flower colours [Nc]	0.85	0.75 – 0.97	**0.012**	1.18	1.04 – 1.33	**0.010**	1.28	1.13 – 1.45	**<0.001**	0.58	0.51 – 0.66	**<0.001**	0.83	0.74 – 0.94	**0.003**	1.03	0.92 – 1.16	0.595	0.57	0.50 – 0.64	**<0.001**
Percentage of yellow flowers [Y< 50%]	0.69	0.51 – 0.93	**0.016**	0.50	0.37 – 0.68	**<0.001**	0.32	0.24 – 0.44	**<0.001**	0.65	0.49 – 0.86	**0.003**	0.50	0.38 – 0.67	**<0.001**	0.40	0.30 – 0.53	**<0.001**	0.33	0.25 – 0.45	**<0.001**
Place of residence [rural areas]	1.62	1.03 – 2.57	**0.038**	2.01	1.14 – 3.53	**0.016**	1.66	0.90 – 3.06	0.105	1.48	0.89 – 2.45	0.132	1.96	1.15 – 3.34	**0.014**	1.43	0.81 – 2.51	0.215	1.22	0.64 – 2.31	0.549
Gender [male]	1.11	0.74 – 1.65	0.615	1.07	0.65 – 1.75	0.788	1.15	0.67 – 1.95	0.612	1.11	0.71 – 1.71	0.655	1.11	0.70 – 1.77	0.659	1.00	0.61 – 1.64	0.994	1.16	0.67 – 2.01	0.606
Percentage of alien plants [A< 50%]	1.92	1.55 – 2.38	**<0.001**	1.22	0.99 – 1.52	0.063	1.20	0.97 – 1.49	0.090	2.05	1.67 – 2.52	**<0.001**	1.45	1.18 – 1.78	**<0.001**	1.28	1.05 – 1.57	**0.016**	0.51	0.41 – 0.63	**<0.001**
σ^2	3.29			3.29			3.29			3.29			3.29			3.29			3.29		
τ00	1.05 person.id			1.79 person.id			2.16 person.id			1.38 person.id			1.59 person.id			1.82 person.id			2.32 person.id		
ICC	0.24			0.35			0.40			0.30			0.33			0.36			0.41		
N	188 person.id			188 person.id			188 person.id			188 person.id			188 person.id			188 person.id			188 person.id		
Marginal R^2 / Conditional R^2	0.229 / 0.415			0.048 / 0.384			0.064 / 0.435			0.296 / 0.504			0.147 / 0.425			0.075 / 0.404			0.160 / 0.507		
